# Gut Microbiota and Acylcarnitine Metabolites Connect the Beneficial Association between Estrogen and Lipid Metabolism Disorders in Ovariectomized Mice

**DOI:** 10.1128/spectrum.00149-23

**Published:** 2023-05-04

**Authors:** Mengmeng Guo, Xi Cao, De Ji, Hui Xiong, Ting Zhang, Yujiang Wu, Langda Suo, Menghao Pan, Daniel Brugger, Yulin Chen, Ke Zhang, Baohua Ma

**Affiliations:** a Key Laboratory of Animal Biotechnology, Ministry of Agriculture, College of Veterinary Medicine, Northwest A&F University, Yangling, China; b Key Laboratory of Animal Genetics, Breeding and Reproduction of Shaanxi Province, College of Animal Science and Technology, Northwest A&F University, Yangling, China; c Institute of Animal Sciences, Tibet Academy of Agricultural and Animal Husbandry Sciences, Lhasa, China; d Institute of Animal Nutrition and Dietetics, Vetsuisse-Faculty, University of Zurich, Zurich, Switzerland; Chengdu University

**Keywords:** estradiol benzoate, gut microbiota, lipid metabolism, ovariectomy, acylcarnitine

## Abstract

Decreased estrogen level is one of the main causes of lipid metabolism disorders and coronary heart disease in women after menopause. Exogenous estradiol benzoate is effective to some extent in alleviating lipid metabolism disorders caused by estrogen deficiency. However, the role of gut microbes in the regulation process is not yet appreciated. The objective of this study was to investigate the effects of estradiol benzoate supplementation on lipid metabolism, gut microbiota, and metabolites in ovariectomized (OVX) mice and to reveal the importance of gut microbes and metabolites in the regulation of lipid metabolism disorders. This study found that high doses of estradiol benzoate supplementation effectively attenuated fat accumulation in OVX mice. There was a significant increase in the expression of genes enriched in hepatic cholesterol metabolism and a concomitant decrease in the expression of genes enriched in unsaturated fatty acid metabolism pathways. Further screening of the gut for characteristic metabolites associated with improved lipid metabolism revealed that estradiol benzoate supplementation influenced major subsets of acylcarnitine metabolites. Ovariectomy significantly increased the abundance of characteristic microbes that are significantly negatively associated with acylcarnitine synthesis, such as *Lactobacillus* and Eubacterium ruminantium group bacteria, while estradiol benzoate supplementation significantly increased the abundance of characteristic microbes that are significantly positively associated with acylcarnitine synthesis, such as *Ileibacterium* and *Bifidobacterium* spp. The use of pseudosterile mice with gut microbial deficiency greatly facilitated the synthesis of acylcarnitine due to estradiol benzoate supplementation and also alleviated lipid metabolism disorders to a greater extent in OVX mice.

**IMPORTANCE** Our findings establish a role for gut microbes in the progression of estrogen deficiency-induced lipid metabolism disorders and reveal key target bacteria that may have the potential to regulate acylcarnitine synthesis. These findings suggest a possible route for the use of microbes or acylcarnitine to regulate disorders of lipid metabolism induced by estrogen deficiency.

## INTRODUCTION

In female mammals, decreased estrogen levels and higher circulating androgen levels lead to the development of metabolic syndrome (1, 2), which primarily includes cardiovascular disease (3) and type 2 diabetes (4). Reduced estrogen levels in postmenopausal women promote changes in the levels of numerous lipids (lipoproteins, apolipoproteins, and triacylglycerols) circulating in blood (5), thus triggering impaired lipid metabolism in the body. Impaired lipid metabolism affects the production of adipocytokines, proinflammatory cytokines, and reactive oxygen species (6), which also play a role in the development of insulin resistance (7), abdominal obesity, and dyslipidemia (8). Several investigations have demonstrated that estrogen not only modulates hepatic cholesterol uptake and reverses cholesterol transport but also regulates cholesterol production in the liver (9–11). In line with this, estrogen mediates typical fat distribution. In particular, hypothalamic estrogen signaling is important in controlling white fat distribution, white fat partitioning, and brown fat thermogenesis (12). These effects on adipose tissue metabolism are predominantly achieved via the activation of estrogen receptor alpha (ER) in neurons (13). Estrogen also interacts with other hormones and adipokines, such as leptin, growth hormone-releasing peptide YY (PYY), and insulin, to influence disorders of lipid metabolism (12).

Gut commensal bacteria can produce and secrete hormones, and the interaction between microbes and hormones affects host metabolism, immunity, and behavior. It has been demonstrated that the abundance of gut microbiota is significantly influenced by estrogen and that gut microbiota composition also significantly influences estrogen levels (14). The gut microbiome affects estrogen levels in the host by secreting β-glucuronidase, an enzyme that releases bound estrogen. This enables estrogen binding to the estrogen receptor and facilitates subsequent downstream physiological effects (15). Reduced β-glucuronidase activity leads to a decrease in estrogen in circulation and altering of estrogen receptor activation, thus leading to low estrogen-induced diseases such as obesity, metabolic syndrome, cardiovascular disease, and cognitive decline (16). The “estrobolome,” a group of genes in the gut microbiota that encode enzymes that metabolize estrogen, is regulated by estrogen (17). To prevent harmful inflammation and malfunctioning of mitochondria, estrogen-related receptors (ESRRA) maintain gut homeostasis through activation of autophagy and regulation of gut microbiota (18). Research on postmenopausal women revealed that their gut microbiota are less diverse than those of premenopausal women. The postmenopausal gut microbiome lacks the short-chain fatty acid (SCFA)-making *Faecalibacterium* and *Roseburia* species as well as mucolytic *Akkermansia* (19–21). Studies on gut microbiota of people with polycystic ovarian syndrome (PCOS) caused by estrogen deprivation have revealed that Bacteroides vulgatus is markedly more prevalent and is correlated with a decline in glycine deoxycholic acid and taurodeoxycholic acid levels. Transplanted fecal microbiota from PCOS-afflicted women or Bacteroides vulgatus into recipient mice resulted in infertility, insulin resistance, altered bile acid metabolism, decreased interleukin 22 (IL-22) production, and ovarian dysfunction (22). In ovariectomized (OVX) mouse models, estrogen deficiency reduces the abundance of *Bacillus* spp. and *Akkermansia* while simultaneously increasing serum cholesterol, hepatic lipogenesis, and adipogenic gene expression. These observations indicate that estrogen plays an important role in obesity and glucose and lipid homeostasis by regulation of the gut microbiota (23, 24).

The uterus is the target organ for the biological effects of estrogen, with its function, metabolism, and cell proliferation being regulated and influenced by estrogen (25). Studies have shown that the weight of the uterus can directly reflect the estrogen concentration in the body (26). OVX mice have also been shown to be a proper model system for studying estrogen insufficiency (27). Estradiol benzoate (EB) as a more attractive estrogenic medication can metabolize in the liver and convert to estradiol. Estradiol is then transported to the target tissue and is bound to the estrogen receptor to provide estrogenic effects through transcription and protein synthesis (28). Since EB influences female human health and livestock reproduction, numerous efforts have been made to study the effects of EB therapy on the regulation of reproductive performance, intestinal inflammation, and metabolic disorders during menopause in animals (29–31). However, it is still ambiguous as to how changes in gut microbial composition relate to disturbances in lipid metabolism brought on by insufficient female estrogen production. It is even more intriguing to test whether various doses of EB supplementation could help abnormal lipid metabolism in OVX mice by affecting gut bacteria and their metabolites. To address this question, OVX mice and pseudo-germfree OVX mouse models were used to investigate the effects of EB supplementation on lipid metabolism, gut microbiota, and metabolites using 16S rRNA gene sequencing, transcriptomics, and metabolomics. Our results reveal an important role for gut microbes and their metabolites in the regulation of estrogen deficiency-mediated disorders of lipid metabolism.

## RESULTS

### High-dose estradiol benzoate supplementation alters growth phenotypes and serum hormone levels in OVX mice.

**FIG 1:**
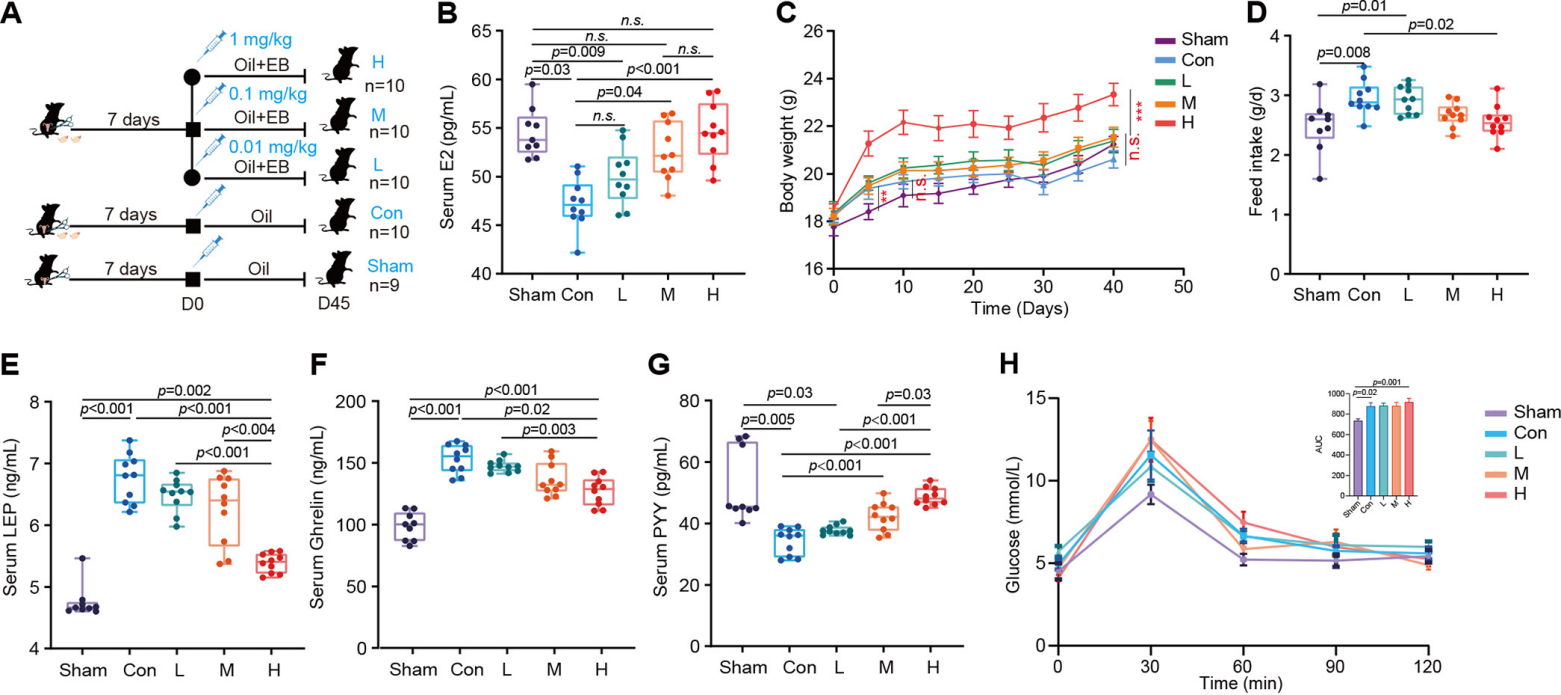
Effects of subcutaneous EB supplementation on growth phenotypes and serum hormone levels of OVX mice. (A) Design paradigm. (B) Serum E2 concentration. n.s., not significant. (C) Mouse body weight versus time curves. (D) Feed intake. (E to G) Concentrations of serum LEP (E), ghrelin (F), and PYY (G). (H) Curves of blood glucose levels during OGTT and AUC. Data differences in mouse subjects were analyzed by one-way ANOVA with Tukey’s test. Sham (*n* = 9), ovariectomy surgery was performed, preserving both ovaries; Con (*n* = 10), ovaries were surgically removed on both sides; L (*n* = 10), injections of 0.01 mg/kg EB in OVX mice; M (*n* = 10), injections of 0.1 mg/kg EB in OVX mice; H (*n* = 10), injections of 1 mg/kg EB in OVX mice.

We exposed groups of OVX mice to various EB concentrations (0.01 [group L], 0.1 [group M], and 1 [group H] mg/kg) (Fig. 1A) to assess the effects of EB on the physiology and serum hormone levels. The subcutaneous EB injection dose was found to be directly proportional to the serum estradiol concentration (Fig. 1B). The estradiol concentrations in the M and H groups significantly increased in comparison to those in the control (Con) group but did not differ significantly from those in the sham-treated (Sham) group (see Materials and Methods for a description of the Con and Sham groups) (*P* > 0.05) (Fig. 1B). However, low doses of EB had no discernible effect on blood estrogen levels (*P* > 0.05) (Fig. 1B). A large number of previous studies have shown a significant increase in body weight in mice after ovariectomy when administered high-fat diets and a gradual decrease in body weight upon external estrogen supplementation (32–34). In contrast, under low-fat diet conditions, the OVX group gained weight faster than the Sham group after 7 days (*P* = 0.008) (Fig. 1C). However, the difference between the two groups was no longer significant after 7 days (*P* > 0.05) (Fig. 1C), with the H group gaining weight noticeably faster than the Con group from day 7 to day 45 (*P* < 0.001) (Fig. 1C). The H group consumed much less food than the Con group (*P* = 0.008) (Fig. 1D), while the Con group consumed significantly more food than the Sham group (*P* = 0.02) (Fig. 1D). Serum leptin (LEP), ghrelin, and PYY concentrations were measured to further test whether EB can alter the gastrointestinal hormones in OVX mice and subsequently affect feed intake. The results revealed a gradient decrease in LEP and ghrelin concentrations and a gradient increase in PYY concentration with increasing EB dose (Fig. 1E to G). Thus, supplementation of high doses of EB significantly increased body weight and progressively restored gastrointestinal hormone-like alterations in OVX mice.

### High-dose estradiol benzoate supplementation reduces lipid accumulation in OVX mice.

**FIG 2:**
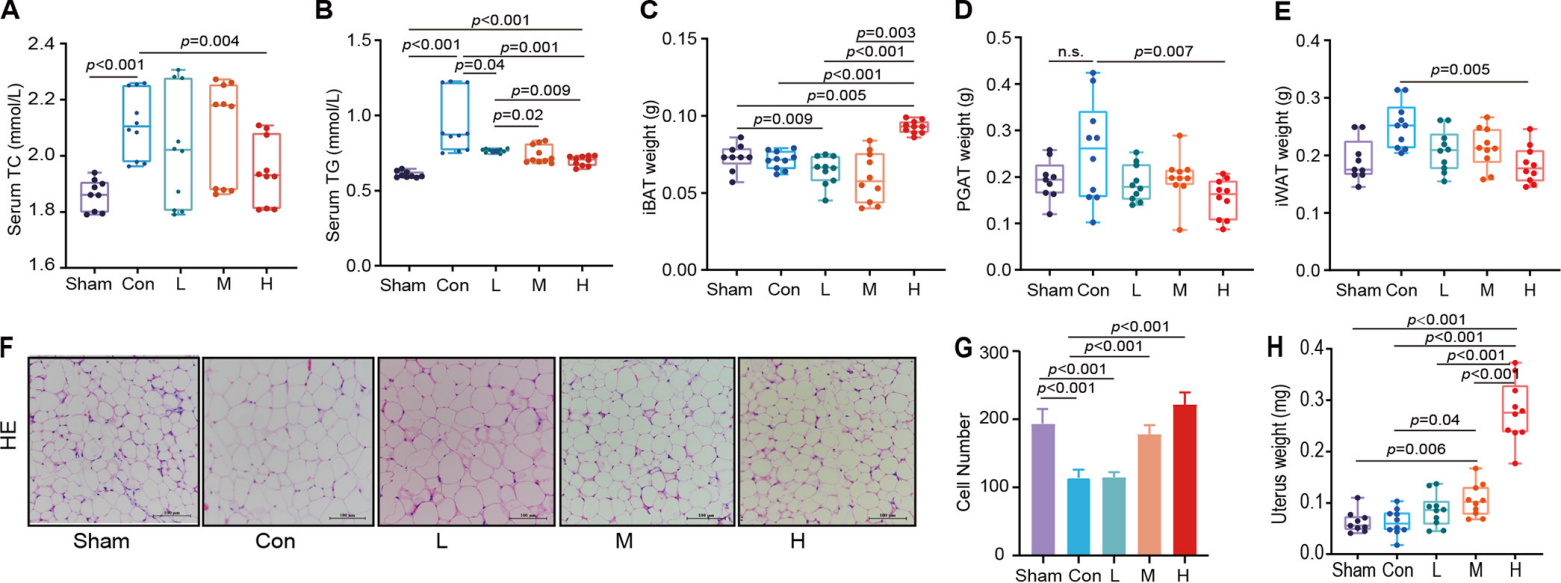
Effects of high-dose subcutaneous EB supplementation on lipid accumulation in OVX mice. (A and B) Serum TC and TG concentrations. (C to E) iBAT (C), PGAT (D), and iWAT (E) weights. (F) Microscopic visualization of histological staining (×200 magnification; scale bar, 100 μm) of PGAT (*n* = 9). (G) Mean cell number of adipocytes calculated from at least three discontinuous scans in nine mice from each group. (H) Weight of the uterus.

High doses of EB did not have a significant effect on the liver total cholesterol (TC) and triglyceride (TG) concentrations (see Fig. S1A and B in the supplemental material) but significantly reduced the free fatty acid (FFA) concentrations (*P* = 0.04) (Fig. S1C). High EB dose decreased serum lipids in OVX mice, as evidenced by a significantly lower level of TC and TG in the EB intervention groups and a gradient decrease in serum TG concentration in OVX mice with increasing EB dose (Fig. 2A and B). Hematoxylin and eosin (H&E) staining showed no severe cytoplasmic vacuolation or swelling in the liver of the OVX group, suggesting that OVX does not cause hepatotoxicity under low-fat diet conditions (Fig. S1D). Additional examination of the weights of brown fat and white adipose fat showed that in OVX mice, high doses of EB significantly increased brown fat (iBAT) content and decreased white adipose tissue (iWAT) and perigonadal adipose tissue (PGAT) contents (Fig. 2D and E). In addition, OVX mice showed lower glucose tolerance than Sham group mice, while EB treatment did not have any effect on glucose tolerance in comparison with Con individuals (Fig. 1H). The number of adipocytes per unit area was greatly increased, while the size of adipocytes was reduced in OVX mice treated with medium and high doses of EB (Fig. 2F and G). Unsurprisingly, the uterine weight increased gradually with increasing EB concentration (*P* < 0.001) (Fig. 2H). Interestingly, blood levels of proinflammatory factors (IL-1β, IL-6, tumor necrosis factor alpha [TNF-α], and monocyte chemotactic protein 1 [MCP-1]) decreased with increasing EB dose (Fig. S1E to H). This demonstrated that high doses of EB result in decreased fat accumulation while increasing brown fat deposition and decreasing the elevation of OVX-related serum proinflammatory markers.

### Estradiol benzoate supplementation alters the transcriptome profile of the liver in OVX mice.

Estrogen is indirectly associated with the liver and regulates lipid metabolism in a significant way (35). Therefore, the effect of EB therapy on the liver transcriptome in OVX mice was examined in detail. We analyzed the RNA sequencing (RNA-seq) data from liver tissues from the Sham, Con, L, M, and H (*n* = 6) groups. RNA-seq-based transcriptome profiling generated a total of 1,459,419,084 raw reads that eventually gave a total of 1,411,466,810 clean reads after quality control. Of these, the percentage of base error rate (Q30) ranged between 92.48% and 94.76%, with an average GC content of 49.20%. These observations suggested that the RNA-seq data obtained were of good quality.

**FIG 3:**
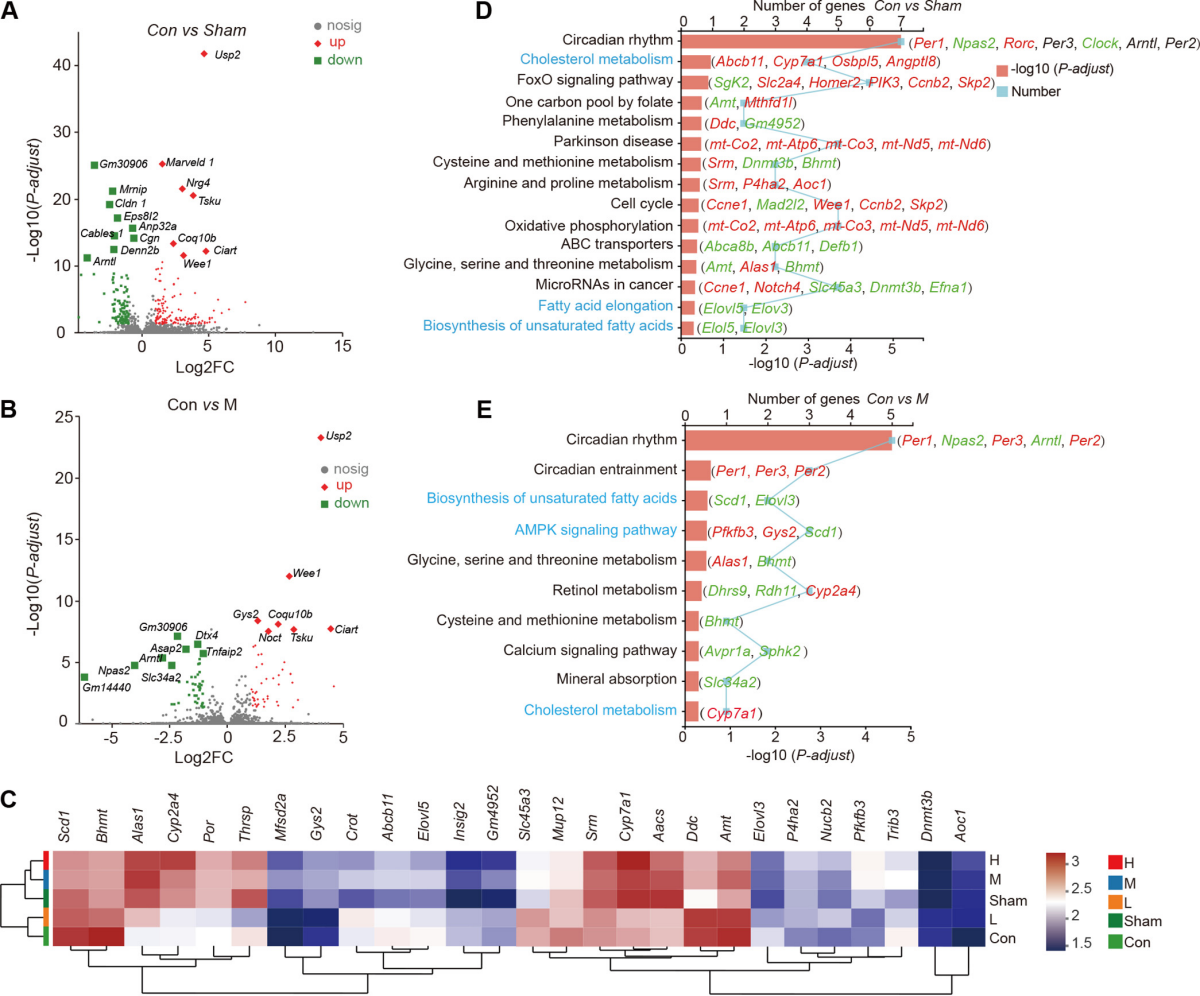
Effects of subcutaneous EB supplementation on the hepatic transcriptome profile of OVX mice. (A and B) Volcano plots of RNA-seq analyses of Con versus Sham groups (A) and Con versus M groups (B). Red diamonds represent upregulated DEGs; green diamonds represent downregulated DEGs. (C) Heat maps of DEGs expressed in the five groups. (D and E) KEGG enrichment analyses of Con versus Sham groups (D) and Con versus M groups (E). The gene names to the right of the red bar represents the DEG involved in the corresponding pathway. Gene names in red represent upregulated DEGs; gene names in green represent downregulated DEGs.

**FIG 4:**
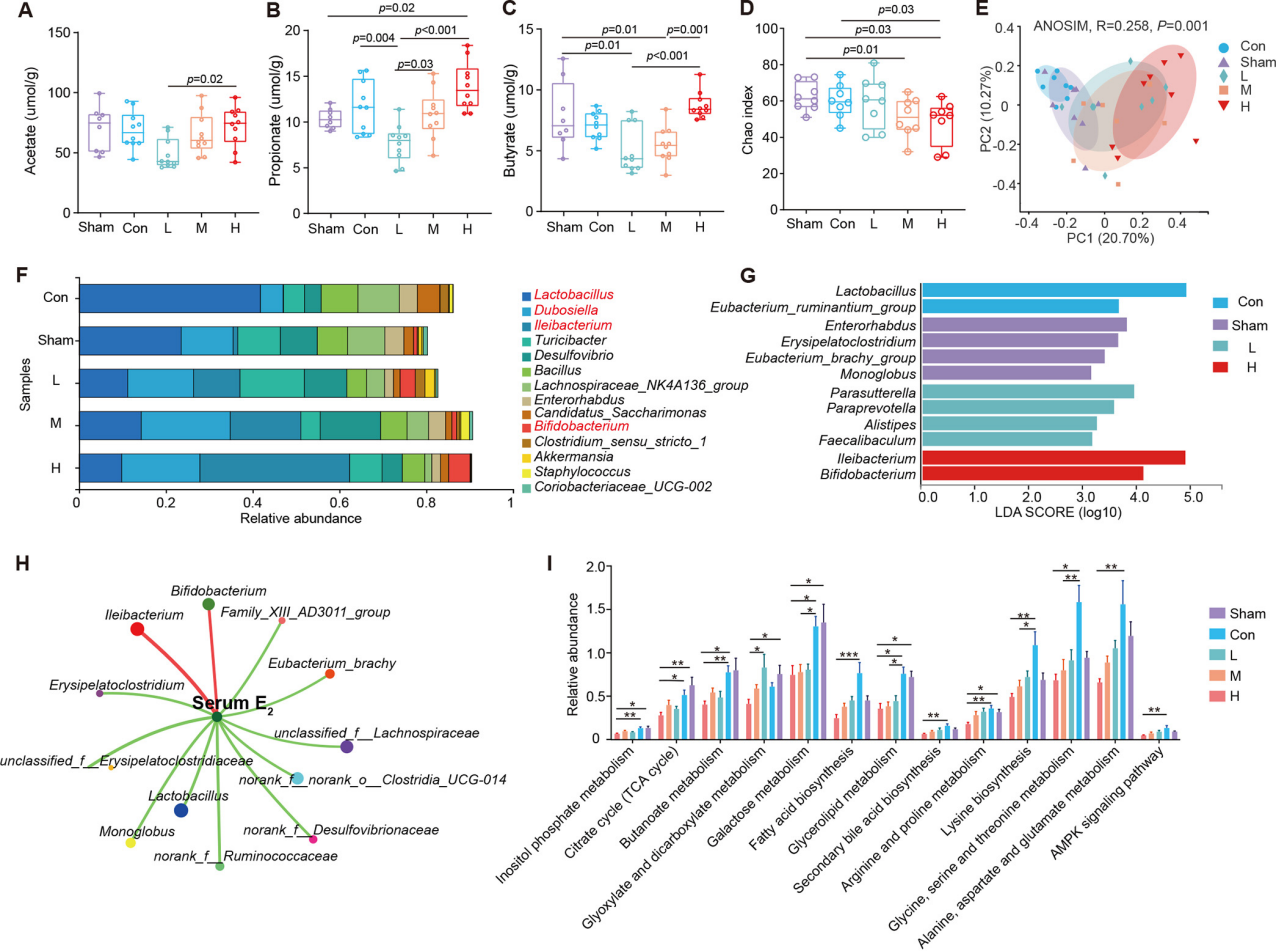
Effects of subcutaneous EB supplementation on cecal microbiota and functionality in OVX mice. (A to C) Contents of the microbial fermentation products acetate (A), propionate (B), and butyrate (C) in the cecum. Differences in data were assessed by one-way ANOVA with Tukey’s test. (D) Alpha-diversity at the ASV level in five groups. (E) PCoA plots based on the ASV matrix of cecal microbiota in five groups. Beta-diversity by ANOSIM analysis. (F) Relative abundances of cecal microbiota at the genus level in five groups. (G) Differential bacterial genera selected by LEfSe analysis with an LDA score of >3.0 in cecal microbiota. (H) Spearman correlation network between different microbial and serum E2 levels of samples among the five groups. The line colors ranged from green (negative correlation) to red (positive correlation) at an absolute ≥ 0.6 and *P* < 0.05 level of significance. (I) Prediction and assessment of potential functional pathways of cecal content microbiota based on PICRUSt2. Differences in data were assessed by one-way ANOVA with Tukey’s test. *, *P* < 0.05, and **, *P* < 0.01.

We defined differentially expressed genes (DEGs) as those with an adjusted *P* value (*P*-adjust) of <0.05 and an absolute log2 fold change of ≥1. A total of 345 DEGs were found in the Sham, L, M, and H groups in comparison to the Con group, with 142, 14, 49, and 97 upregulated genes (see Fig. 4A and B; Fig. S2A and B) and 97, 11, 41, and 77 downregulated genes (see Fig. 4A and B; Fig. S2A and B), respectively. Detailed information about each DEG is shown in Table S2. In the livers of OVX mice given medium to high doses of EB, 65 DEGs were identified in comparison to the Con group (Table S2), with the majority being *USP2*, *ALAS1*, *CYP2A4*, *POR*, *THRSP*, *CYP7A1*, *SRM*, *AACS*, *TSKU*, *NAMPT*, *RAB36*, *TRMT9B*, *WEE1*, *COQ10B*, *CIART*, *NPAS2*, and *SLC5A6* (Fig. 3C). Significant alterations were also seen in various cellular signaling pathways in members of the Sham group. Cholesterol metabolism (*Abcb11*, *Cyp7a1*, *Osbpl5*, *Angptl8*), fatty acid elongation (*Elovl5*, *Elov3*), and biosynthesis of unsaturated fatty acids were the key pathways connected to the DEGs (Fig. 3D). Several biological signaling pathways, including those involved in progesterone-mediated oocyte maturation, IL-17 signaling, estrogen signaling, and the MAPK signaling pathway (involved in *STMN1*), were significantly altered in the L group (Fig. S2C). Several biological signaling pathways, such as the AMPK signaling system (involved in *HMGCR*, *PFKFB3*, *GYS2*, and *SCD1*) and the production of unsaturated fatty acids (involved in *ELOVLL5* and *SCD1*), had significant modifications in the M and H group (Fig. 3E; Fig. S2D). These findings indicate that medium to high doses of EB changed the expression of genes and pathways involved in cholesterol metabolism and unsaturated fatty acid synthesis in the liver of OVX mice.

### Estradiol benzoate supplementation alters cecum microbiota composition and potential function in OVX mice.

We further investigated the effects of EB treatment on gut microbiota composition and function in OVX mice and the correlation between the altered gut microbes and host lipid metabolism. SCFAs that are metabolites of microbiota were quantified to determine their concentrations in the ceca of mice. Low-dose EB treatment significantly decreased the concentrations of propionate (*P* = 0.004) (Fig. 4A to C), while high-dose EB treatment increased butyrate and propionate concentrations. However, the differences were not statistically significant owing to large individual differences in replicates (*P* > 0.05) (Fig. 4A to C). We then used bacterial 16S rRNA gene profiling to characterize 49 different cecum samples from the five groups and to examine their bacterial diversity. Based on the Chao index, the H group generally had less alpha-diversity at the amplicon sequence variation (ASV) level than the Con group (*P* = 0.03) (Fig. 4D). According to the Sob, Shannon, and Simpson indices, there was no significant change in alpha-diversity at the ASV level between the groups (*P* > 0.05) (Fig. S3A). Principal-coordinate analysis (PCoA) at the ASV level based on the Bray-Curtis dissimilarity and Jaccard indexes showed a clear separation of microbial community structure between the five groups (analysis of similarity [ANOSIM], *P* = 0.001) (Fig. 4E; Fig. S3B). We also analyzed variations in microbial communities between the five groups at the genus level and found that the abundance of *Lactobacillus* spp. was significantly lower in the Sham, L, M, and H groups than in the Con group. In contrast, the L, M, and H groups significantly outnumbered the Con group in terms of the abundance of *Dubosiella*, *Ileibacterium*, and *Bifidobacterium* spp. (*P* < 0.001) (Fig. 4F). Using linear discriminate analysis effect size (LEfSe) analysis to identify additional key signature bacteria, we discovered that OVX mice had significantly higher levels of *Lactobacillus* and Eubacterium ruminantium species bacteria and that high doses of EB treatment led to significantly higher levels of *Ileibacterium* and *Bifidobacterium* spp. (Fig. 4G).

To ascertain if the imbalance in cecal microbiota has any correlation with the lipid metabolism and hormonal alterations in OVX mice, we evaluated the correlation between blood estradiol (E2) levels, metabolic phenotype, and differential microbiota. *Bifidobacterium* spp. (*r *= 0.63, *P = *0.001) and *Ileibacterium* spp. (*r *= 0.49, *P < *0.001) abundances were significantly correlated with E2 (Fig. 4H). Additionally, we discovered a positive correlation between iBAT weight and *Ileibacterium* spp. (*r* = 0.31, *P = *0.04) and *Bifidobacterium* spp. (*r* = 0.43, *P = *0.005) (Fig. S3). It is worth noting that based on PICRUST2-predicted microbial function, high doses of EB treatment significantly reduced the pathway expression of butanoate metabolism, fatty acid biosynthesis, glycerolipid metabolism, galactose metabolism, secondary bile acid biosynthesis, and AMP-activated protein kinase (AMPK) signaling pathway in the ceca of OVX mice (Fig. 4I). These results demonstrated that high doses of EB altered the microbiota makeup in OVX mice and decreased enrichment of lipid metabolism functional pathways in cecal bacteria.

### Estradiol benzoate supplementation induces accumulation of acylcarnitine in OVX mouse ceca.

**FIG 5:**
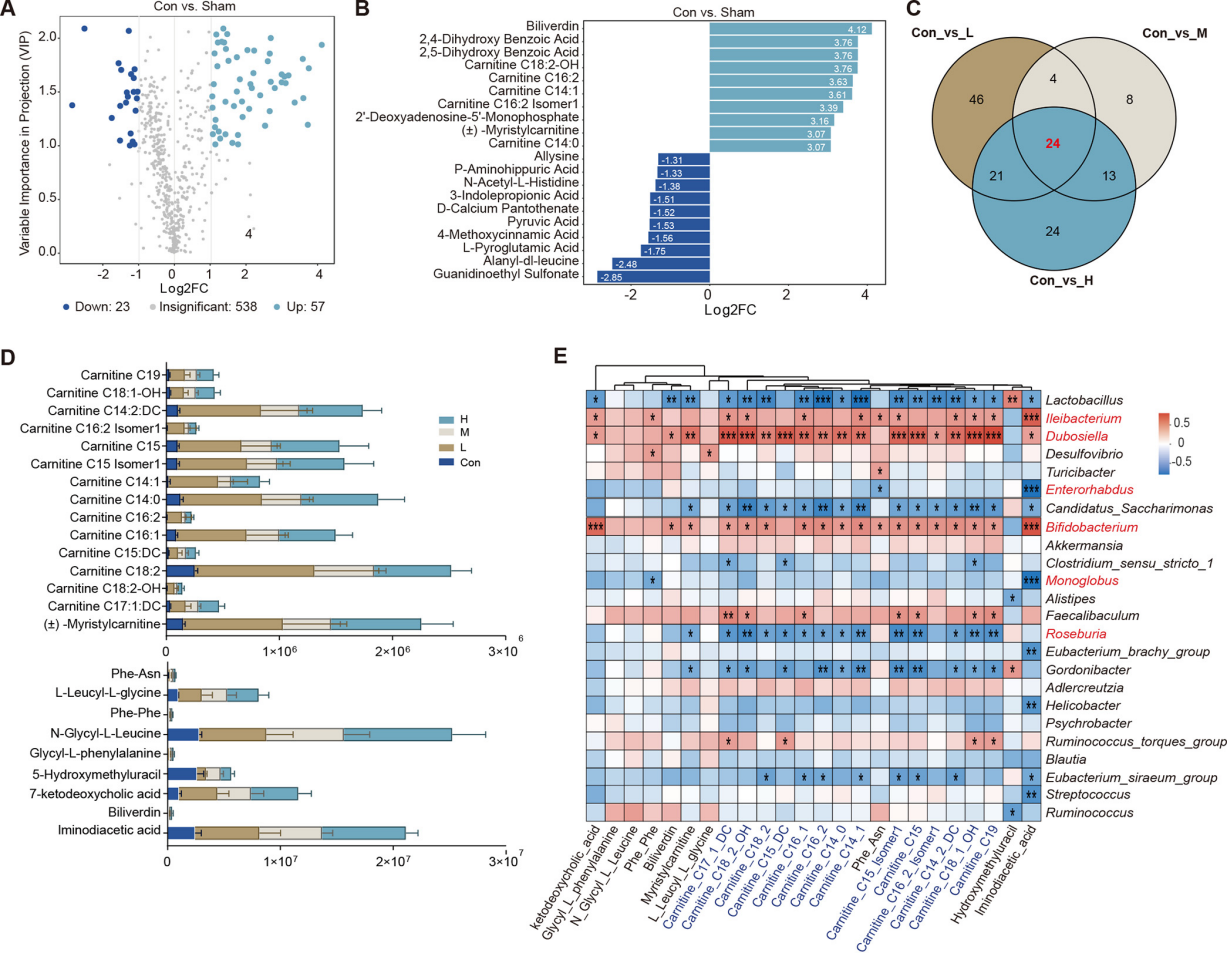
Effects of subcutaneous EB supplementation on cecal acylcarnitine in OVX mice. (A) Volcano plots of metabolite analyses of Con versus Sham groups. (B) Differential metabolite bar graphs of Con versus Sham groups; the top 10 metabolites are indicated after log_2_ processing of the differences in abundance multiples of metabolites between the two groups. (C) Venn diagram of differential metabolites in Con versus L, Con versus M, and Con versus H groups. (D) Relative abundances of 24 common differentially expressed metabolites in four groups. (E) Correlation heat maps of differentially expressed metabolites and different microbial genera among the five groups. Color denotes positive (red) and negative (blue) correlation at an absolute ≥ 0.6 and *P* < 0.05 level of significance. *, *P* < 0.05, **, *P* < 0.01, and ***, *P* < 0.001.

A widely targeted metabolomics technique based on ultraperformance liquid chromatography-tandem mass spectrometry (UPLC-MS/MS) was employed to examine the metabolites in OVX mouse ceca to ascertain the effects of EB supplementation on the metabolic profile. We defined differentially expressed metabolites (DEs) as those with a fold change of ≥2 and ≤0.5. A total of 78, 95, 49, and 82 DEs were discovered in the Sham versus Con, L versus Con, M versus Con, and H versus Con groups, respectively (Fig. 5A; Fig. S4A to C). Of these, 57, 56, 39, and 62 DEs were upregulated in the Sham, L, M, and H groups, respectively (Fig. 5A; Fig. S4A to C). These findings show that acylcarnitine levels in OVX mice (Con) were significantly lower than those in the Sham group. This was especially true for carnitine C14:0, carnitine C16:2 isomer 1, carnitine C14:1, carnitine C16:2, and carnitine C18:2-OH (Fig. 5B). We discovered 24 distinct metabolites that were differentially expressed in the EB addition gradients after using Venn plots to identify coupregulated metabolites in the L, M, and H groups (Fig. 5C). These mainly included carnitine C19, carnitine C14:2:DC, carnitine C16:2 isomer 1, carnitine C15 isomer 1, carnitine C14:1, carnitine C14:0, carnitine C16:2, carnitine C16:1, carnitine C15:DC, carnitine C18:2, carnitine C18:2-OH, and carnitine C17:1:DC (Fig. 5D). Upon administration of EB, a number of additional metabolites, primarily iminodiacetic acid, biliverdin, 7-ketodeoxycholic acid, 5-hydroxymethyluracil, glycyl-l-phenylalanine, *N*-glycyl-l-leucine, and Phe-Phe, were also significantly upregulated (Fig. 5D). Additionally, we highlighted the top 10 DEs for each EB dose in comparison to the Con group (Fig. S4D to F).

We further determined the relationship between the core cecal microbiota and carnitine content upon EB treatment. The abundance of acylcarnitine was positively correlated with *Ileibacterium*, *Dubosiella*, *Bifidobacterium*, and *Faecalibaculum* spp. (*r* > 0.6, *P* < 0.05) (Fig. 5E) and negatively correlated with *Lactobacillus*, *Roseburia*, and “*Candidatus* Saccharimonas” spp. (*r* < −0.6, *P* < 0.05) (Fig. 5E) based on Spearman correlation analysis. A crucial cofactor in lipid metabolism, carnitine facilitates fatty acid entry into mitochondria for oxidative breakdown (36). The alterations in acylcarnitine levels upon EB administration in OVX mice provided additional evidence that host lipid metabolism was profoundly impacted by changes in gut microbiota and their metabolites.

### Estradiol benzoate supplementation alters the serum metabolic profile of OVX mice.

**FIG 6:**
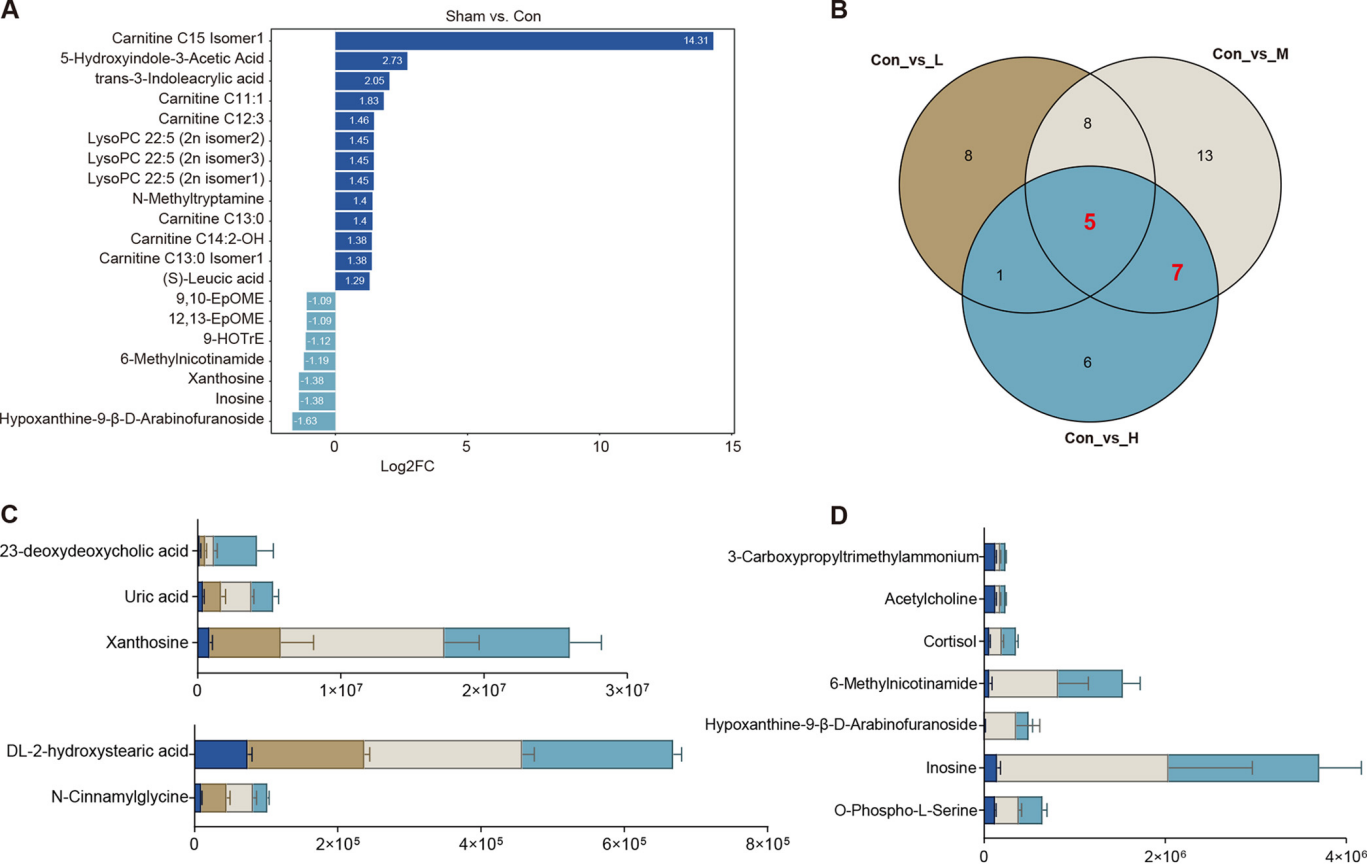
Effects of subcutaneous EB supplementation on the serum metabolic profile in OVX mice. (A) Differential metabolite bar graphs of Con versus Sham groups in serum samples; top 10 metabolites following log_2_ processing of the differences in abundance of multiple metabolites between the two groups. (B) Venn diagram of differential metabolites in Con versus L, Con versus M, and Con versus H groups in serum samples. (C) Relative abundances of five common differential expression metabolites in Con versus L, Con versus M, and Con versus H groups in serum samples. (D) Relative abundances of seven common differential expression metabolites in Con versus M and Con versus H groups in serum samples.

We performed further analysis of the effects of EB treatment on serum metabolic components of OVX mice. In comparison to the Con group, we discovered a total of 22, 33, and 19 DEs in the L, M, and H groups (Fig. S5A to C), respectively, of which 20, 26, and 14 were upregulated DEs, respectively (Fig. S5A to C). We also found that OVX mice had significantly elevated serum acylcarnitine metabolites, with elevated carnitine C15 isomer 1, carnitine C11:1, carnitine C11:1, carnitine C14:2-OH, carnitine C13:0, and carnitine C13:0 isomer 1 levels (Fig. 6A). However, no significant changes were found in carnitine analogs in the serum of OVX mice supplemented with EB (Fig. S5D to F). After further Venn plot analysis to examine coupregulated metabolites in the L, M, and H groups in comparison to the Con group, we discovered five distinct metabolites that were differentially expressed in the three EB gradients (Fig. 6B). These mainly included 23-deoxydeoxycholic acid, uric acid, xanthosine, dl-2-hydroxystearic acid, and *N*-cinnamylglycine (Fig. 6C). We also discovered seven distinct metabolites that were coupregulated in the M and H groups relative to the Con group (Fig. 6B), mainly including 3-carboxypropyltrimethylammonium, acetylcholine, cortisol, 6-methylnicotinamide, hypoxanthine-9-β-d-arabinofuranoside, inosine, and *O*-phospho-l-serine (Fig. 6D).

### Gut microbiota alter the lipid metabolism and synthesis of acylcarnitine in OVX mice supplemented with EB.

**FIG 7:**
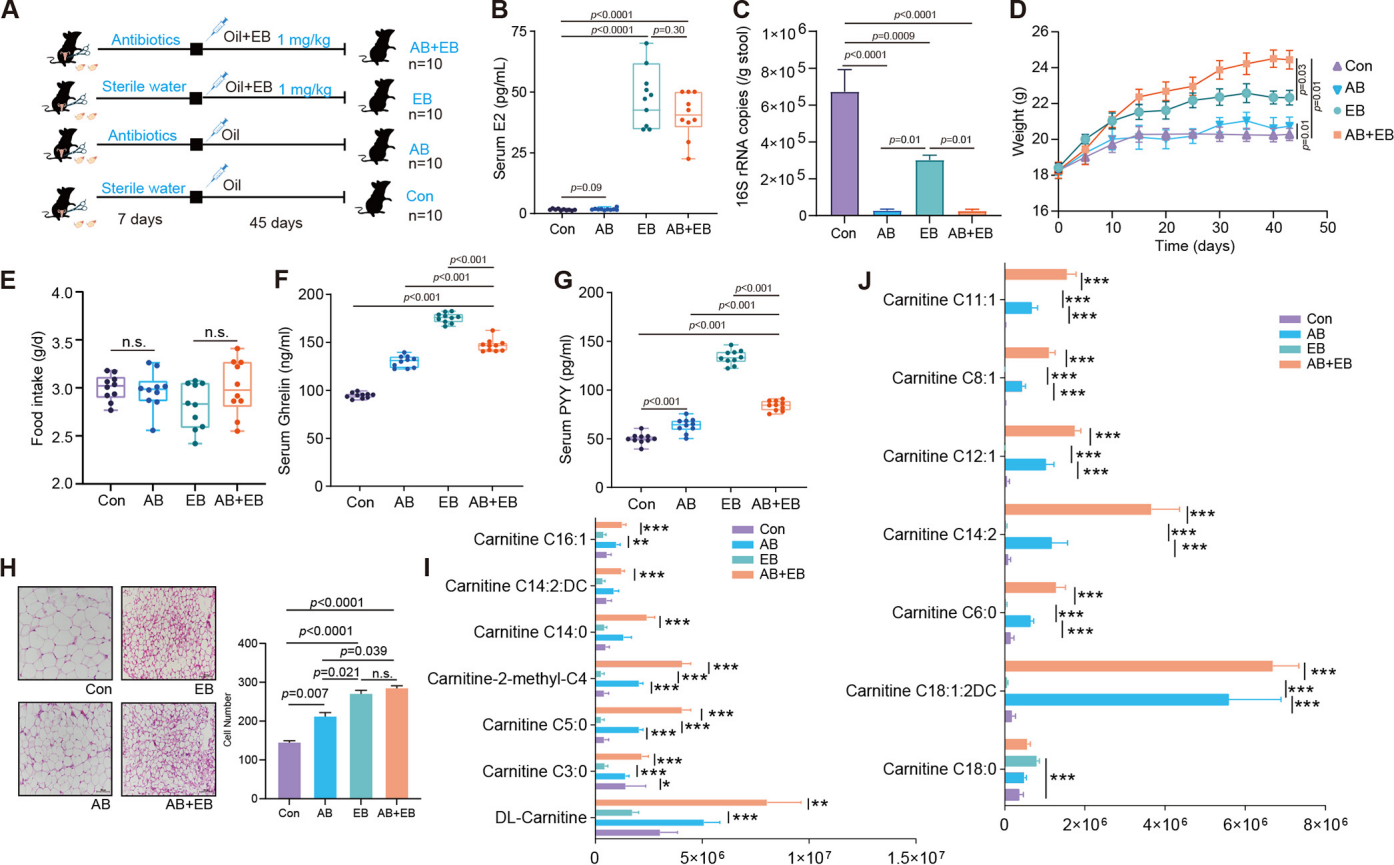
Gut microbiota alterations of the metabolic phenotype of OVX mice in response to subcutaneous EB supplementation. (A) Design paradigm. (B) Serum E2 concentrations. (C) Bacterial density as measured using quantitative PCR of 16S rRNA. (D) Curves of body weight of mice with time. (E) Feed intake. (F and G) Serum ghrelin (F) and PYY (G) concentrations. (H) Microscopic visualization of histological staining (×40 magnification; scale bar, 100 μm) of PGAT (*n* = 4); the mean cell area of adipocytes was calculated from at least three discontinuous scans of nine mice from each group. Differences in data obtained from mouse subjects were assessed by one-way ANOVA with Tukey’s test. (I and J) Relative abundances of differentially expressed metabolites in Con, AB, EB, and AB+EB groups. *, *P < *0.05; **, *P < *0.01; and ***, *P < *0.001.

We constructed a pseudo-germfree mouse model by treating OVX mice with antibiotics. We then administered 1 mg/kg of EB to these mice, and the relevant indices were measured after 45 days of treatment (Fig. 7A). This allowed us to more thoroughly confirm the role of gut microbiota in lipid regulation of EB-supplemented OVX mice. We found that there was no discernible difference in serum estradiol levels between the mouse groups treated with EB or with antibiotics plus EB (AB+EB) (see Materials and Methods) (*P* = 0.30) (Fig. 7B). After EB treatment, the abundance of bacteria in the gut was significantly lower than that in the Con group (*P* = 0.0009) (Fig. 7C). Furthermore, administration of antibiotics also significantly removed bacteria from the gut (Fig. 7C). The AB+EB group demonstrated considerably higher weight gain than the EB-only group (*P* = 0.03) (Fig. 7D), although feed consumption did not change significantly (Fig. 7E). We also found lower serum ghrelin and PYY concentrations in the AB+EB group than in the EB group (*P* < 0.001) (Fig. 7F and G) and also significantly higher serum concentrations of the gastrointestinal hormone LEP (*P* = 0.002) (Fig. S6K). In addition, there was no significant difference in number of white adipocytes per unit area or size of adipocytes between the two groups (*P > *0.05) (Fig. 7H). However, the iBAT, PGAT, and iWAT weights were considerably lower in the AB+EB group than in the EB group (*P* < 0.05) (Fig. S6M to O). Additionally, there were no appreciable differences in the blood and hepatic TC, TG, and FFA concentrations between the AB+EB and EB groups (*P* > 0.05) (Fig. S6A, B, and H to J). AB treatment did not have any discernible effects on the change in uterine weight in EB-treated mice (*P* > 0.05) (Fig. S6C). However, the serum levels of IL-1β, TNF-α, and MCP-1 in mice of the AB+EB group were noticeably greater than those in mice of the EB group (*P* < 0.05) (Fig. S6D to G).

We further examined metabolite changes in the gut and found that EB treatment significantly increased the abundance of carnitine C18:0 (Fig. 7J). However, in germfree mice, we found that EB treatment promoted the production of acylcarnitine in the gut and that the presence of microbiota inhibited the production of acylcarnitine components (Fig. 7J), mainly dl-carnitine, carnitine C5:0, carnitine C8:1, carnitine C6:0, carnitine C18-1:2DC, carnitine C14:2, carnitine C12:1, and carnitine C11:1. Taken together, our results indicate that gut microbial deficiency significantly increases acylcarnitine synthesis and also greatly alleviates lipid metabolism disorders in OVX mice.

## DISCUSSION

In this study, we investigated the effects of different doses of EB on the lipid metabolism disorder caused by estrogen deficiency by using the OVX mouse model. We screened key metabolites that alleviate the lipid metabolism disorder in OVX mice by using metabolomics. Our results are preliminary and reveal the effect of gut microbiota on the production of the key metabolites. We report that OVX mice have significantly altered body weight and gastrointestinal hormone concentrations upon high-dose EB administration, which affects lipid metabolic function. We found that these altered metabolic phenotypes were linked to gut microbes. Interestingly, removal of gut microbes worsened EB supplementation-induced weight gain but decreased white fat deposition in mice. Additionally, we found that the critical gut bacteria *Bifidobacterium* and *Ileibacterium* spp. play crucial roles in the lipid metabolic phenotype. Our findings provide a crucial theoretical foundation for EB therapy of animals with ovariectomies or metabolic illnesses brought on by estrogen deprivation as a result of ovarian malfunction.

Previous studies have shown that EB inhibits ovariectomy-induced weight gain in rats upon subcutaneous implantation of 17-estradiol extended-release tablets (0.25 mg/pill, 60-day release) (32, 33). EB inhibits post-OVX weight gain in female mice fed a high-fat diet when given 0.3 mg/kg of oral EB daily (34). A study of 30-week-old OVX mice fed a high-fat diet (45% energy as fat with extra fat from lard) or low-fat diet (10% energy as fat) for 11 weeks found that there was a significant increase in body weight in the high-fat diet group, while the difference was not significant in the low-fat diet group (37). Other studies have shown that withdrawal of estrogen through OVX increases food intake and decreases motor activity, a phenomenon known to occur in rodents and humans (38). However, this weight gain can be observed only after 1 month of surgery (39). In this study, we found that dorsal subcutaneous EB injection at low and medium doses while on a low-fat diet did not significantly alter body weight in OVX mice. In contrast, EB injection at high doses (1 mg/kg/3 days) resulted in significantly increased body weight. This increase was maintained from day 7 to day 45 after treatment in the study cohort and did not occur by chance. We speculate that EB supplementation slows the rate of gastric emptying and the overall metabolic rate in OVX mice (40).

Estrogen-mediated regulation of body weight and feed intake in animals is associated with changes in serum gastrointestinal hormones (41). Estrogen administration (oral or transdermal estrogen treatment for 6 months) has previously been shown to increase plasma gastric hunger hormone concentrations as well as increased serum leptin levels (42), which is consistent with our findings. A previous study indicates that ovariectomy results in decreased exercise activity and energy expenditure and causes glucose intolerance and obesity (43). It also suggests that E2 can enhance the effects of leptin on energy balance by regulating body weight, fat distribution, and energy expenditure, as well as increasing leptin sensitivity and cooperating with leptin to regulate body weight, food intake, and energy expenditure (44). On a mechanistic level, estrogen reduces leptin resistance and enhances p-STAT3 signaling in the hypothalamus arcuate nuclei via the estrogen receptor alpha (45). In general, E2 increases leptin sensitivity through its anorexigenic effect, which is the main reason for the reduced feed intake in the high-dose EB group.

Using a mouse model of ER deficiency in hepatocytes, it was previously demonstrated that the ability of estrogen to reduce hepatic steatosis is lost when ER is lacking in the liver, indicating that estrogen can physiologically regulate hepatic lipid metabolism via ER (9, 46, 47). Estradiol therapy prevents the effects of hyperinsulinemia on lipid metabolism in OVX female mice via inhibition of adipogenesis, lowering of hepatic TG, and improving insulin action on glucose metabolism (32). However, we did not notice any significant changes in the liver of OVX mice treated with EB. EB supplementation significantly decreased TG and TC concentrations in the liver of these mice when gut microbiota were removed, suggesting that estrogen regulation of hepatic lipid physiology metabolism is associated with gut microbiota. Previous research has also supported the relationship between host gut microbiota and lipid homoeostasis, which is highly important for health (48). We establish that the gut microbes of OVX mice differ significantly from those of the Sham group mice and that EB supplementation further modifies gut microbial composition in OVX mice. The genera *Ileibacterium* and *Bifidobacterium* were markedly enriched in the high-dose EB group. *Bifidobacterium* spp. are closely linked to lipid metabolism of the body, according to earlier studies (49). Enzymes from *Bifidobacterium* spp. can dissociate and decompose bile acid salts, thus degrading the bile acid that returns to the intestine through the hepatic and intestinal circulation (50). *Bifidobacterium* spp. can also produce hydroxymethylglutaric acid (HMG), which affects the activity of HMG coenzyme A reductase in cholesterol (51). Additionally, it has been demonstrated that Bifidobacterium longum APC1472 and Bifidobacterium breve APC6331 lessen the internalization signaling of the gastric starvation hormone-regulated receptor (52). Patients with PCOS who received supplements containing *Bifidobacterium* V9 had significantly lower levels of luteinizing hormone and follicle-stimulating hormone and significantly higher levels of sex hormone and intestinal SCFAs (53). Less functional research has been done on *Ileibacterium* spp., but in large-scale sequencing data, *Ileibacterium* spp. were found to be closely related to the regulation of lipid metabolism (54, 55). However, the primary functional mechanism of action of *Ileibacterium* needs to be further examined.

A previous study has demonstrated the importance of carnitine as a coenzyme in lipid metabolism and the role it plays in mitochondrial lipid metabolism. Levels of acylcarnitine, a by-product of carnitine metabolism, can reveal the relationship between fatty acid oxidation and mitochondrial stress in young animals (56). In the present study, we found that the cecal acylcarnitine content was significantly increased and the serum acylcarnitine content was significantly decreased when OVX mice were supplemented with EB. Acylcarnitine accumulation in the serum and cecum is an essential diagnostic marker for mitochondrial fatty acid oxidation diseases (57, 58). Previous research has shown that acylcarnitine concentrations in the feces of germfree mice are higher than those in the feces of conventionally fed mice (57). These observations are consistent with our study, where we found that the increased acylcarnitine content in the cecum and the decreased serum acylcarnitine content in OVX mice are due to changes in the microbial composition of the intestine caused by OVX treatment that weaken the ability of intestinal epithelial cells to absorb acylcarnitine (59, 60). Changes in serum acylcarnitine levels can influence hepatic lipid metabolism and, as a result, white fat accumulation in mice. We verified the plausibility of this hypothesis by using compound antibiotic-treated OVX mice (AB group). We also found that increased acylcarnitine content was considerably favorably associated with the abundance of *Ileibacterium*, *Dubosiella*, and *Bifidobacterium* spp. and significantly negatively correlated with the abundance of *Roseburia* and *Lactobacillus* spp. A previous study has demonstrated that the colon has high levels of the carnitine transporter protein (Octn2), which is involved in the uptake of carnitine and acylcarnitine (61). It has also been demonstrated that if the bacterial community in the colon reaches a high cell density, the population-sensing system can facilitate intercellular communication within the bacterial community by producing molecules such as spore-stimulating factors and lipopolysaccharide (LPS) (61). These factors can regulate gene expression in colonic cells. Spore-stimulating factors compete with acylcarnitine for Octn2 transport, leading to inhibition of acylcarnitine uptake (62). Conversely, bacterial LPS molecules activate Toll-like receptor 4 (TLR4) in colonocytes, which stimulates PPAR-γ activity and Octn2 gene expression, leading to increased carnitine or acylcarnitine uptake (63). This further confirms that changes in gut microbiota have an impact on the expression of carnitine transporter proteins in the intestine, which has an impact on acylcarnitine absorption. Thus, acylcarnitine concentrations in serum have a significant impact on the body's capacity to control fat deposition and metabolic function.

### Conclusion.

Taken together, our findings demonstrate that EB supplementation can alter the gut microbiome and effectively alleviate lipid metabolism disorders caused by ovariectomy. Our finding that an EB supplementation influences major subsets of acylcarnitine metabolites highlights the importance of gut microbes to greatly reduce the concentration of acylcarnitine in the intestine. Furthermore, EB supplementation promotes the synthesis of acylcarnitine in the intestine, alleviating lipid accumulation in the animal. We thus propose that acylcarnitine can be used as a therapeutic agent to prevent disorders of lipid metabolism for subsequent applications and research.

## MATERIALS AND METHODS

### Ethical approval.

All experimental procedures involving animals were approved by the Ethics Committee of Northwest A&F University (approval numbers 20210507–009 and 20211107–005).

### Animals, diets, and experimental design.

The mice were kept in individual metal cages at room temperature (23 ± 1°C) with a 12-h light/12-h dark cycle and ready access to autoclaved water and normal chow (Xietong, Jiangsu) (see Table S1 in the supplemental material), The low-fat diet utilized in this experiment was the open-source, purified AIN-93G diet (4.00 kcal/g) that comprises 64.3%, 15.8%, and 20.3% of the total kilocalories from carbohydrate, fat, and protein, respectively. A total of 49 7-week-old female C57BL/6J specific-pathogen-free mice were randomly separated into five groups after 1 week of acclimation. The groups were designated Sham (*n* = 9), Con (*n* = 10), L (0.01 mg/kg of body weight EB), M (0.1 mg/kg of body weight EB), and H (1 mg/kg of body weight EB) (*n* = 10) (64). Both ovaries of the mice in the Con, L, M, and H groups were surgically removed; mice in the Sham group underwent the same surgical procedure, but the ovaries were left intact (65). After a week of healing from surgery, mice in the Sham and Con groups received subcutaneous injections of 100 μL sesame oil every 4 days, while mice in the other groups received injections of 100 μL EB (HPLC grade, ≥98%; Yuanye Bio-Technology, Shanghai, China) at the designated concentrations once every 4 days. EB was dissolved in sesame oil as a solvent. The body weight and feed intake of mice were recorded once every 4 days. All mice were sampled by dissection after 45 days.

A total of 40 7-week-old female C57BL/6J specific-pathogen-free mice were randomly separated into four groups after undergoing bilateral ovariectomies following 1 week of acclimation. The groups were Con (*n* = 10), antibiotics (AB; *n* = 10), EB (*n* = 10), and AB+EB (*n* = 10). Antibiotic mixtures were provided in drinking water to the OVX mice in the AB and AB+EB groups to eliminate gut microbiota. An antibiotic mixture consisting of 1 g/L ampicillin, 1 g/L streptomycin, 1 g/L gentamicin, and 0.5 g/L vancomycin (Macklin, Shanghai, China) was constituted in autoclaved water (66). Mice in the Con and EB groups were given normal sterile water. EB injections of 1 mg/kg were given to the mice in the EB group and the AB+EB group once every 4 days. Body weight and feed intake were also recorded once every 4 days. All mice were sampled via dissection after 45 days.

### Histopathology of PGAT and liver.

Fresh PGAT and liver tissues (Yike Biotechnology Co., Xi'an, China) from mice were fixed with 4% paraformaldehyde, embedded in paraffin, sectioned into 4-mm-thick pieces, and stained with H&E. Adipocyte sizes in PGAT sections were measured using Image-Pro Plus v6.0 (Media Cybernetics, Inc., Silver Spring, MD, USA), and the mean adipocyte cell area was calculated by using at least three discontinuous scans from nine mice in each group. The liver segment histologic scores were evaluated as previously described (67).

### Measurement of serum physiological index.

Blood samples from mice were collected and stored at room temperature for 40 min. The samples were then centrifuged for 15 min at 3,500 rpm at 4°C. The supernatant was collected and stored for future investigation at −80°C. The levels of serum TC, TG, E2, and glucose (Glu) were measured using HY-50061, HY-50062, HY-10029 and HY-50063 kits (Huaying, Beijing, China) in an automatic biochemical analyzer (model 7160; Hitachi, Tokyo, Japan) according to the instructions provided by the manufacturer. Leptin (LEP) and PYY were measured using HY-10075 and HY-10184 radioimmunoassay kits (Huaying, Beijing, China), and ghrelin was measured using ELISA kits in accordance with the manufacturer’s instructions (Huaying, Beijing, China). Serum levels of IL-1β, IL-6, IL-10, TNF-α, and MCP-1 were measured using ELISA kits in accordance with the manufacturer’s instructions (Huaying, Beijing, China).

### OGTT.

All mice were given glucose (2 g/kg body weight) by oral gavage after 8 h of fasting. Blood samples were taken from the tip of the tail vein at 0, 30, 60, 90, and 120 min post-glucose load to measure the concentrations of glucose by use of a blood glucose meter (Accu-Chek Performa; Roche, USA). The results of the oral glucose tolerance test (OGTT) blood glucose were plotted and analyzed as the area under the curve (AUC) from 0 to 120 min (68).

### Hepatic lipid measurement.

Approximately 0.10-g portions of semithawed liver samples were homogenized in equivalent amounts (1/9) of homogenizing buffer (pH 7.4; 0.01 mol/L) and added to screw-cap tubes containing 0.1 mmol/L EDTA-Na_2_, 0.8% NaCl, and Tris-HCl. The tubes were then incubated twice in ice-cold water for 5 min, followed each time by shaking for 5 min at 25 Hz/s with a TissueLyser. The supernatant was then collected after centrifugation of the samples for 10 min at 2,500 rpm at 4°C. The levels of hepatic total cholesterol, triglycerides, and FFA were then tested using A111-1-1, A110-1-1, and A042-1-1 assay kits from Jiancheng Bioengineering (Jiancheng, Nanjing, China). The findings from the hepatic lipid test were normalized to the total protein concentration in accordance with the manufacturer’s instructions (Jiancheng, Nanjing, China).

### Analysis of cecum content SCFAs.

For each cecal content sample, 0.03 g of cecal content was weighed into a 2-mL centrifuge tube, to which 400 μL of ultrapure water was then added. The mixture was then homogenized and incubated for 30 min. The samples were then centrifuged at 10,000 rpm for 10 min at 4°C, and 300-μL volumes of supernatants were aspirated. The supernatants were mixed with 30 μL of metaphosphoric acid before being incubated at 4°C for 3 to 4 h. To remove contaminants from the protein samples, centrifugation was carried out at 13,500 rpm for 15 min at 4°C. An equal volume of transcrotonic acid was then added to the supernatants. A 0.22 μm water phase filter membrane was used to separate the cecal material from the supernatant after the mixture was incubated for 20 min. The supernatants were then stored in 2-mL screw-cap vials. An Agilent 7820A gas chromatograph (Agilent Technologies, Santa Clara, USA) was used to measure the SCFA concentrations in the samples (69).

### Cecal bacterial community determination and analysis.

From each of the five groups, a total of 49 cecal lumen samples were taken, instantly frozen in liquid nitrogen, and stored at 80°C until use. The E.Z.N.A. stool DNA kit (Omega Bio-Tek, Norcross, GA, USA) was used to extract total DNA from the cecum lumen contents per the manufacturer's instructions. The concentration and purity of DNA samples were assessed using a Nanodrop 2000 UV-VI spectrophotometer (Thermo Scientific, Wilmington, DE, USA). The quality of isolated DNA was evaluated by electrophoresis on a 1% agarose gel. The V3-V4 region of DNA was amplified using primers 338F (5′-ACTCCTACGGGAGGCAGCAG-3′) and 806R (5′-GGACTACHVGGGTWTCTAAT-3′) on a thermocycler PCR machine (Gene Amp 9700; ABI, USA) (69). Purified amplicons were pooled in equimolar ratios and subjected to paired-end sequencing (2 × 300 bp) using an Illumina MiSeq platform (Illumina, San Diego, CA, USA) and Majorbio Bio-pharm Technology Co., Ltd. (Shanghai, China), standard protocols. Sequencing data were demultiplexed using the QIIME2 pipeline, version 2020.2 (70).

After splitting of the MiSeq paired-end reads, the paired-end reads were quality controlled and filtered based on the sequencing quality using FASTP (v0.19.6) (71). The overlap relationship between the paired-end reads was spliced using FLASH (v1.2.7) to obtain the optimized data after quality-controlled splicing. An average of 44,826 reads was utilized as input for the entire sequencing data set. From these, an average of 20,901 clean reads was obtained after filtering and denoising. The average sequence length of reads obtained was 422 bp. ASVs were identified using the DADA2 plugin (72). All species were classified using the Bayes technique and the Silva 138/16S bacterial database with a classification confidence criterion of 0.7.

The Chao index values based on ASVs were assessed in QIIME2's diversity plugin (core-metrics-phylogenetic pipeline) to determine the sequence diversity, and GraphPad Prism 9 was used to display the results. To test the variation in the gut microbiota (core-metrics-phylogenetic pipeline), the Bray-Curtis dissimilarity was assessed across samples and the PCoA based on ASV levels was carried out in QIIME2 via the diversity plugin. The outputs were displayed using the Majorbio Cloud Platform's web platform (https://www.majorbio.com).

### RNA-seq analysis.

Total RNA was extracted from 30 randomly chosen liver tissue samples (*n* = 6) using TRIzol reagent per the manufacturer's instructions. Genomic DNA was removed from the samples using DNase I (TaKaRa Shuzo, Kyoto, Japan). RNA quality was assessed using an Agilent 2100 Bioanalyzer and a Nanodrop 2000 UV-VI spectrophotometer (Thermo Scientific, Wilmington, DE, USA). An RNA-seq transcriptome library was created from 5 μL of total RNA using a TruSeq RNA sample preparation kit (Illumina, San Diego, CA). RNA concentration was measured using a TBS 380 fluorescence spectrophotometer (Promega, Madison, WI, USA). A paired-end RNA-seq sequencing library was sequenced using the Illumina Nova6000 (2 × 150 bp read length).

Raw paired-end reads obtained from RNA-seq were trimmed and controlled using SeqPrep and Sickle with default parameters. A total of 206.56 GB of clean data were obtained, with each sample generating more than 6.09 GB of clean data with >92.48% of Q30 bases. Clean reads were then separately aligned to the Mus musculus reference genome (GenBank assembly library GRCm39) using the orientation mode with TopHat (v2.1.1) software (73). The comparison rates ranged from 94.95% to 97.34%. Library construction and sequencing were performed by Shanghai Majorbio Bio-pharm Technology Co., Ltd. (Majorbio, Shanghai, China). Differential expression analysis between five groups (Sham, Con, L, M, and H) of samples was performed using DESeq2 (74). DEGs with an absolute log_2_ (fold change) of ≥2 and a *P*-adjust of ≤0.05 were considered to be significantly DEGs. A total of 29,803 expressed genes were detected in this analysis, including 28,134 known genes and 1,669 novel genes. In addition, Kyoto Encyclopedia of Genes and Genomes (KEGG) functional-enrichment analysis was performed to identify DEGs that were significantly enriched in metabolic pathways at a *P*-adjust of ≤0.05 compared with the whole transcriptome. Finally, KEGG pathway enrichment analysis was performed using KOBAS (3.0) (75).

### Widely targeted metabolomics analysis of cecal contents and serum.

All cecal samples were thawed on ice for sample preparation and extraction. Fifty milligrams (±1 mg) of each sample was homogenized with 500 μL of ice-cold methanol-water (70%, vol/vol) along with an internal standard. The mixture was vortexed for 3 min, sonicated in a cold-water bath for 10 min, and vortexed again for 1 min. After centrifugation at 12,000 rpm for 10 min at 4°C, 250 μL of supernatant was aspirated and the mixture was further centrifuged at 12,000 rpm for 5 min at 4°C, after which 150 μL of the supernatant was placed in the lining of the matching injection bottle for onboard analysis.

All serum samples were thawed on ice, vortexed for 10 s, and mixed thoroughly. Then, 50 μL of serum was mixed with 300 μL of pure methanol, and the mixture was vortexed for 3 min before being centrifuged at 12,000 rpm for 10 min at 4°C. The supernatant was then collected and centrifuged further for 5 min at 12,000 rpm and 4°C. After 30 min at −20°C, the sample was centrifuged again for 3 min at 4°C at 12,000 rpm, and 150 μL of the supernatant was aspirated for onboard analysis. A liquid chromatography-electrospray ionization-tandem mass spectrometry (LC-ESI-MS/MS) system was used to analyze the cecal contents and serum sample extracts (UPLC, ExionLC AD system; MS, QTRAP system). The analytical conditions were as follows: UPLC, Waters Acquity UPLC HSS T3 C_18_ column (1.8 m, 2.1 mm by 100 mm); column temperature, 40°C; flow rate, 0.4 mL/min; injection volume, 2 L; solvent system, water (0.1% formic acid)-acetonitrile (0.1% formic acid); gradient program, 95:5 (vol/vol) at 0 min, 10:90 (vol/vol) at 10.0 min, 10:90 (vol/vol) at 11.0 min, 95:5 (vol/vol) at 11.1 min, and 95:5 (vol/vol) at 14.0 min. The LC-MS/MS data were processed by the Analyst 1.6.3 software package based on the self-built metabolite database (Metware Biotechnology Co., Ltd., Wuhan, China), including 826 metabolomic features. The mass spectrometry profile of each sample was integrated with MultiQuant software, and the peak area of each peak represented the relative content of the corresponding substance (76, 77). The peak areas detected for each multiple reaction monitoring ion pair in all samples were corrected using MultiQuant software to make the integration parameters (retention time, peak width, and baseline) consistent. Finally, all the chromatographic peak area integration data were exported and saved (76, 77). The KEGG compound database was used to annotate identified metabolites (78). The *P* values for the hypergeometric test were used to determine the significance of the pathways where significantly regulated metabolites were mapped.

### Statistical analysis.

Bar graphs were generated using GraphPad Prism (V9.0; GraphPad, USA). One-way analysis of variance (ANOVA) with Tukey's multiple-comparison test was used via SPSS (V21; IBM, USA). Differences in relative abundance of bacterial taxa between groups were identified using the Kruskal-Wallis H test in the free online Majorbio Cloud Platform (https://www.majorbio.com). The Kruskal-Wallis test with Dunn's multiple comparisons was used to test variations in the microbiome. Microbial components whose sequences were more prevalent in various groups were identified using LEfSe analysis. To determine the impact of each differentially abundant taxon, the nonparametric factorial Kruskal-Wallis rank sum test and linear discriminant analysis (LDA) were used for LEfSe. A microbiota with an LDA score (log 10) of more than 3.0 was considered to have significantly increased numbers. For PCoA analysis, the ANOSIM function in the vegan package in R was used, including different independent variables based on 999 permutations. Bar figures were generated using the Python 2.7 package. Network correlation analysis was performed using R-3.3.1 (stat). Spearman’s correlation coefficient was calculated in R using cor with method = set to “spearman.” Factors were added to the cooccurrence network if the absolute value of the correlation coefficient was greater than 0.6. For analysis of significantly altered compounds of two groups, distribution-independent ranking tests (based on the Wilcoxon test) and sample-wise permutations (default in the samr package) were used to ascertain significance (false discovery rate < 0.05) (79). Significantly regulated metabolites between groups were determined by a variable importance in projection (VIP) of ≥1 and an absolute log2 fold change of ≥1. VIP values were extracted from the OPLS-DA result, which also contained score plots and permutation plots and was generated using the R package MetaboAnalyst R (80). Identified metabolites were annotated using the KEGG Compound database and were subsequently mapped to the KEGG Pathway database. Pathways with significantly regulated metabolites mapped to the database were then fed into MSEA (metabolite sets enrichment analysis) (81), and their significance was determined by a hypergeometric test’s *P* values.

### Data availability.

The 16S rRNA gene sequencing and RNA-seq data are available from the National Center for Biotechnology Information (NCBI) under accession no. PRJNA890465 and PRJNA892781, respectively.
