# Supplementary material for: Gut Microbiota and Acylcarnitine Metabolites Connect the Beneficial Association between Estrogen and Lipid Metabolism Disorders in Ovariectomized Mice
Source: Microbiol Spectr. 2023 May 4;11(3):e00149-23. doi: 10.1128/spectrum.00149-23 (PMC10269676; doi:10.1128/spectrum.00149-23)
Supplement: Supplemental file 1 — Fig. S1 to S6. Download spectrum.00149-23-s0001.pdf, PDF file, 3.1 MB [file spectrum.00149-23-s0001.pdf]

Supplementary Materials for

Gut microbiota and acylcarnitine metabolites connect the beneficial association between estrogen and lipid metabolism disorders in ovariectomized mice

Mengmeng Guo[1†], Xi Cao[2†], De Ji[3†], Hui Xiong[2], Ting Zhang[2], Yujiang Wu[3], Langda Suo[3], Menghao Pan[1], Daniel Brugger[4], Yulin Chen[2], Ke Zhang[2*], Baohua Ma[1*]

[†]These authors contributed equally to this work.

*Correspondence authors.

Email addresses: malab@nwafu.edu.cn (B.M); kezhang@nwafu.edu.cn (K.Z)

This PDF file includes:

Figures S1 to S6

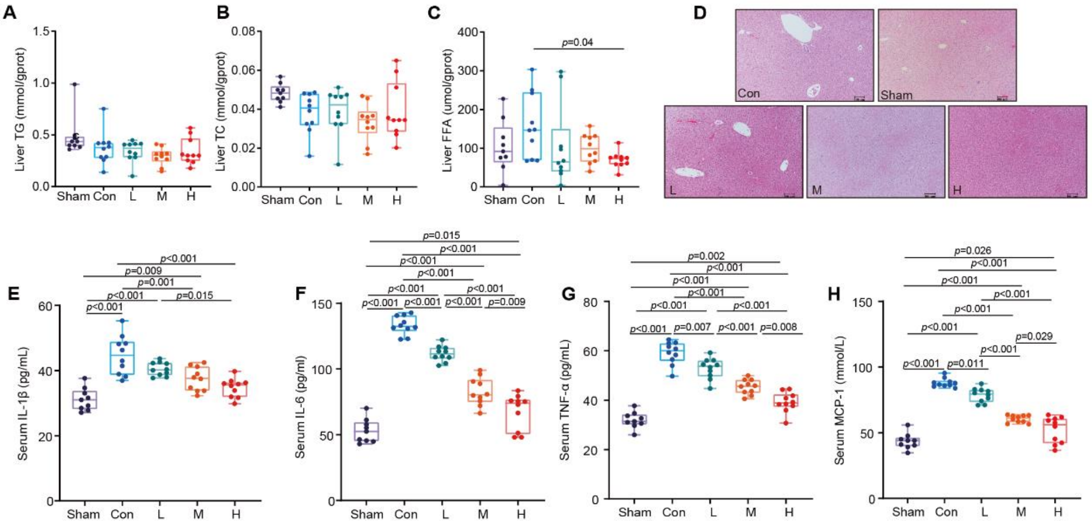

Figure S1. EB supplementation alters hepatic lipid metabolism and reduces serum pro-inflammatory cytokines. (A-C) Concentration of total triglycerides, cholesterol and free fatty acids in the liver, respectively. (D) Representative H&E-stained histological sections of liver (scale bar = 100 μm). (E-H) Serum levels of IL-1β, IL-6, TNF-α, MCP-1.

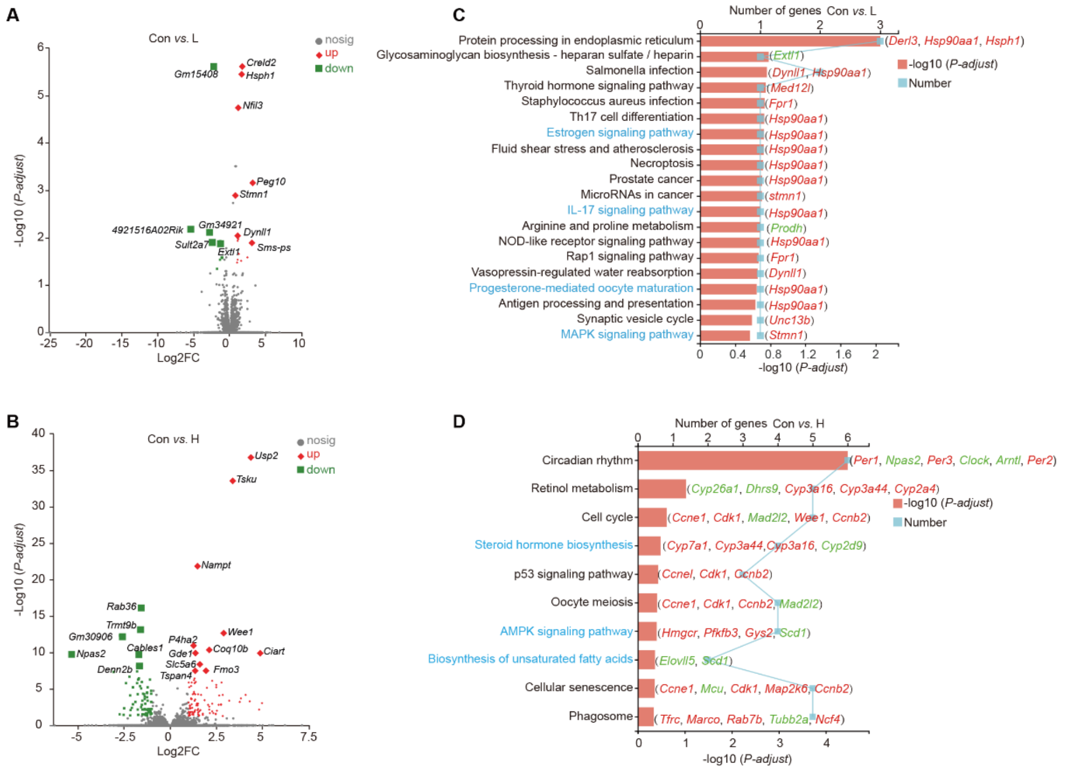

Figure S2. EB supplementation alters the liver transcriptome profile in OVX mice. (A-B) Volcano plots for the RNA-seq analyses of Con vs. L, and Con vs. H groups. Red diamonds represent upregulated DEGs; green diamonds represent downregulated DEGs. (C-D) KEGG enrichment analysis of Con vs. L and Con vs. H groups. The gene names behind each bar represent the gene involved in the pathway. Red font represents upregulated DEGs; green font represents downregulated DEGs.

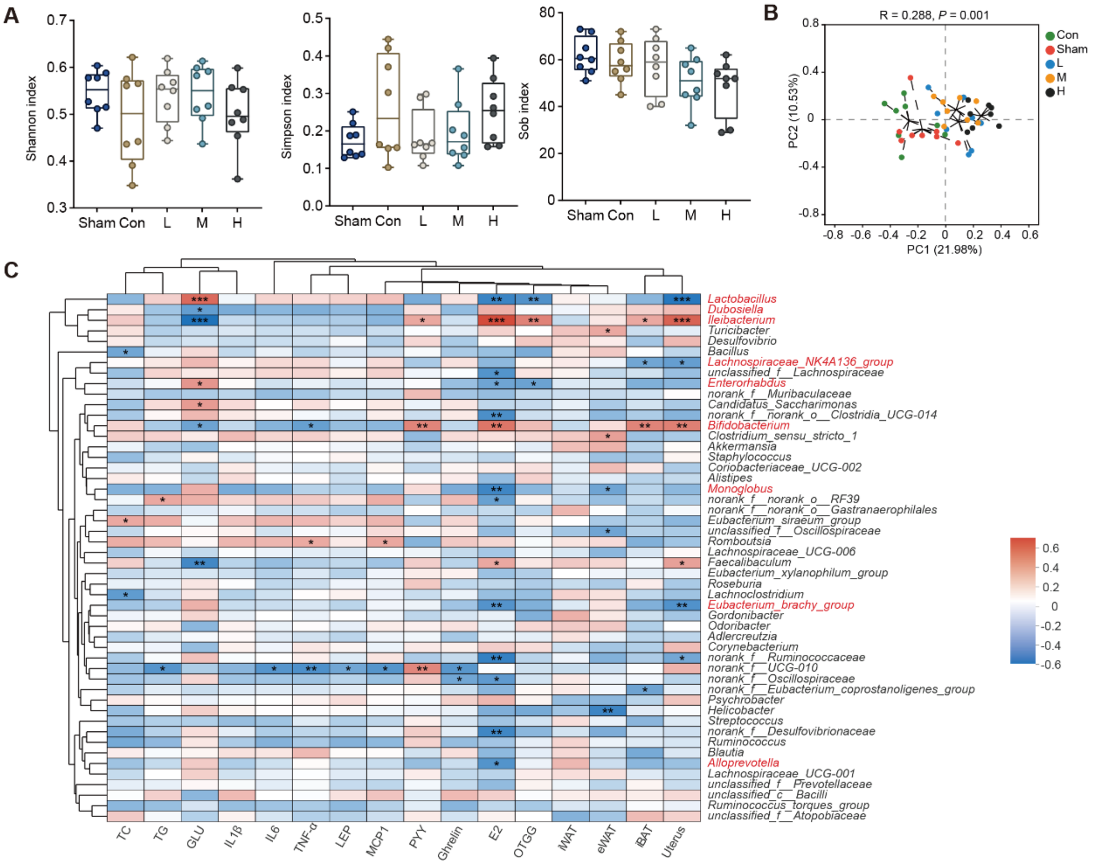

Figure S3. (A) The Shannon, Simpson and Sob index at the ASV level in five groups. (B) PCoA plots in the ASV matrix based on Abund_jaccard of cecal microbiota in five groups. β-diversity by ANOSIM analysis. (C) Heatmap showing the Spearman correlation between phenotype and cecal bacterial abundance in mice at the genus level.

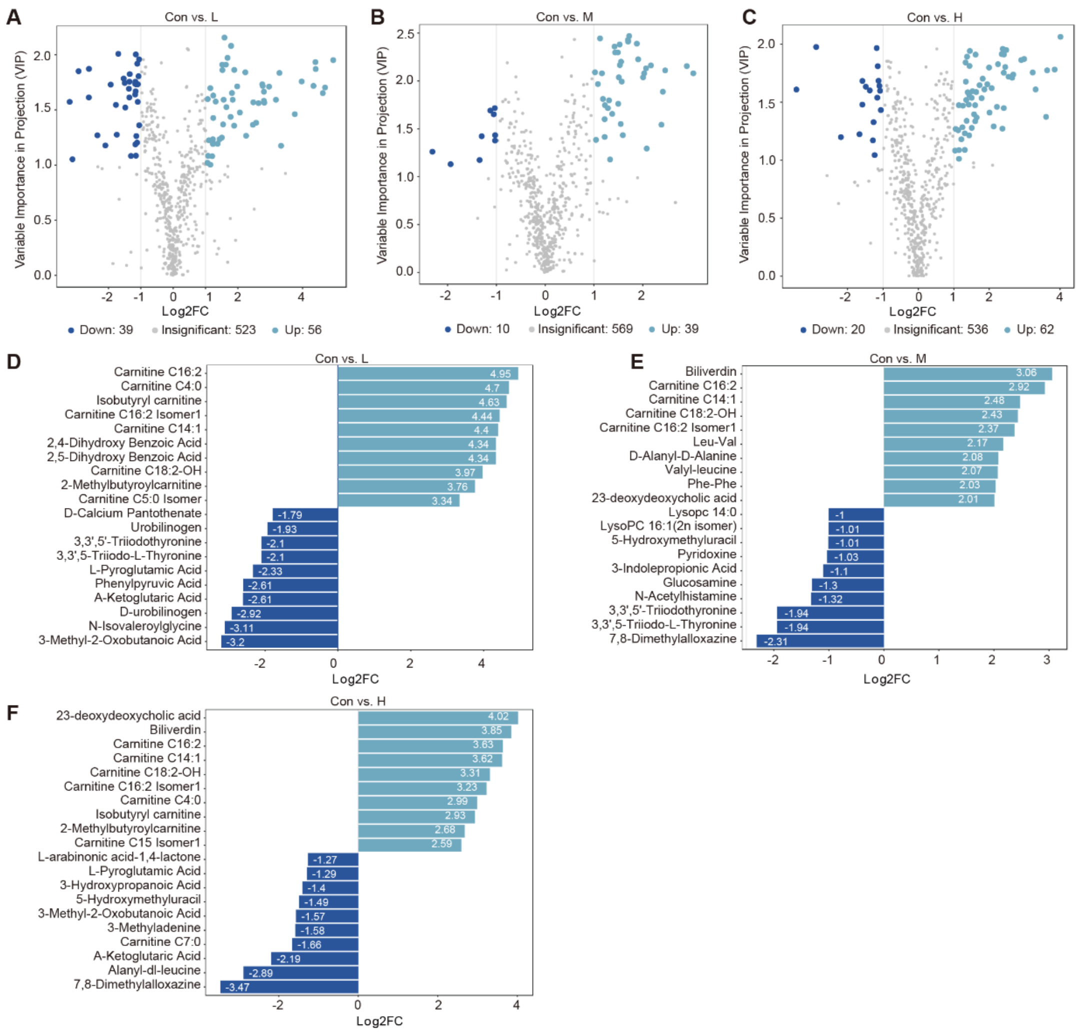

Figure S4. EB supplementation alters the metabolic profile of the cecum content in OVX mice. (A-C) Volcano plots for the metabolic analyses of Con vs. L, Con vs. M and Con vs. H groups. Red diamonds represent upregulated metabolites; green diamonds represent downregulated metabolites. (D-F) Differential metabolite bar graphs of Con vs. L, Con vs. M and Con vs. H in cecum content samples; top 10 metabolites following log2 processing of the difference in abundance multiples of metabolites between the two groups are presented.

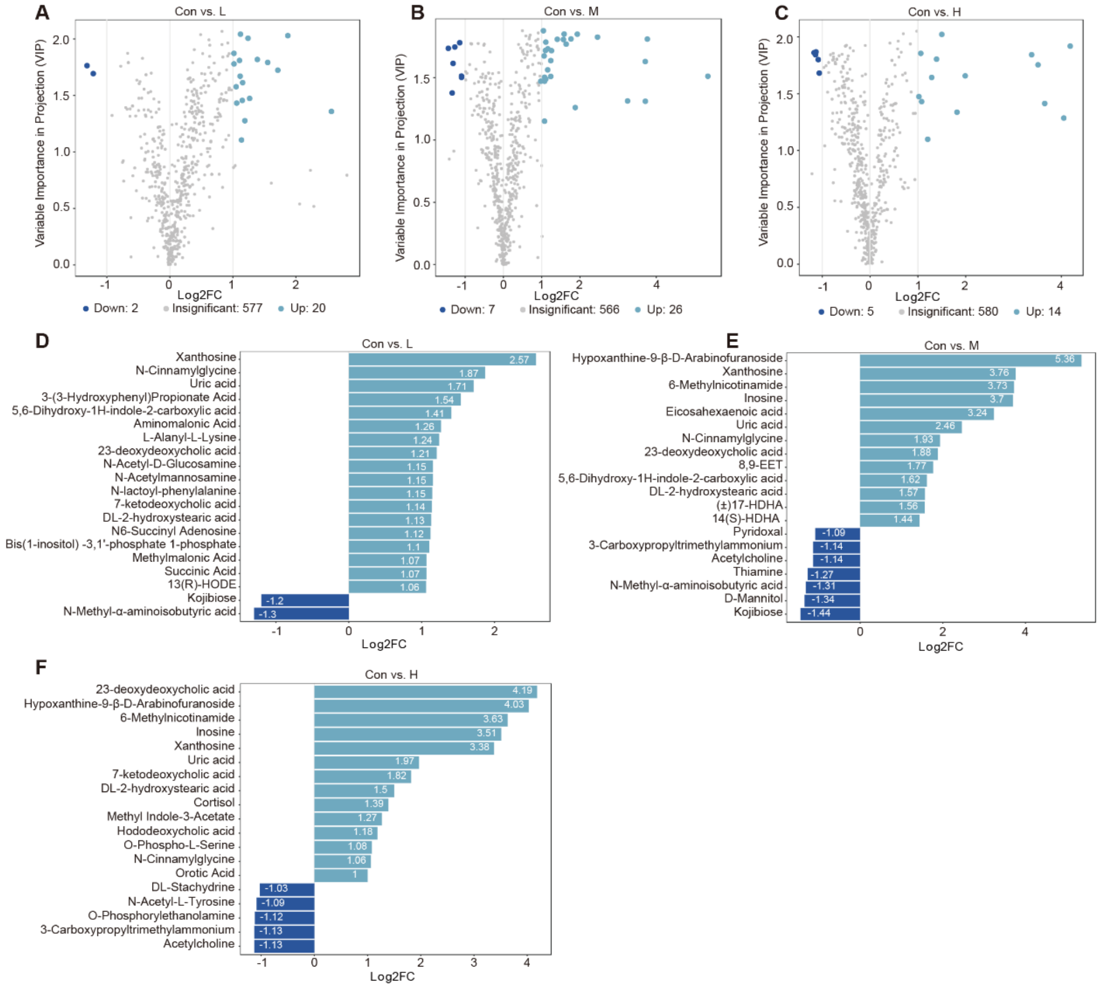

Figure S5. EB supplementation altered the metabolic profile of the serum in OVX mice. (A-C) Volcano plots of metabolic analyses of Con vs. L, Con vs. M and Con vs. H groups. Red diamonds represent upregulated metabolites; green diamonds represent downregulated metabolites. (D-F) Differentially expressed metabolite bar graphs of Con vs. L, Con vs. M and Con vs. H in serum samples; top 10 metabolites following log2 processing of the difference in abundance multiples of metabolites between the two groups are presented.

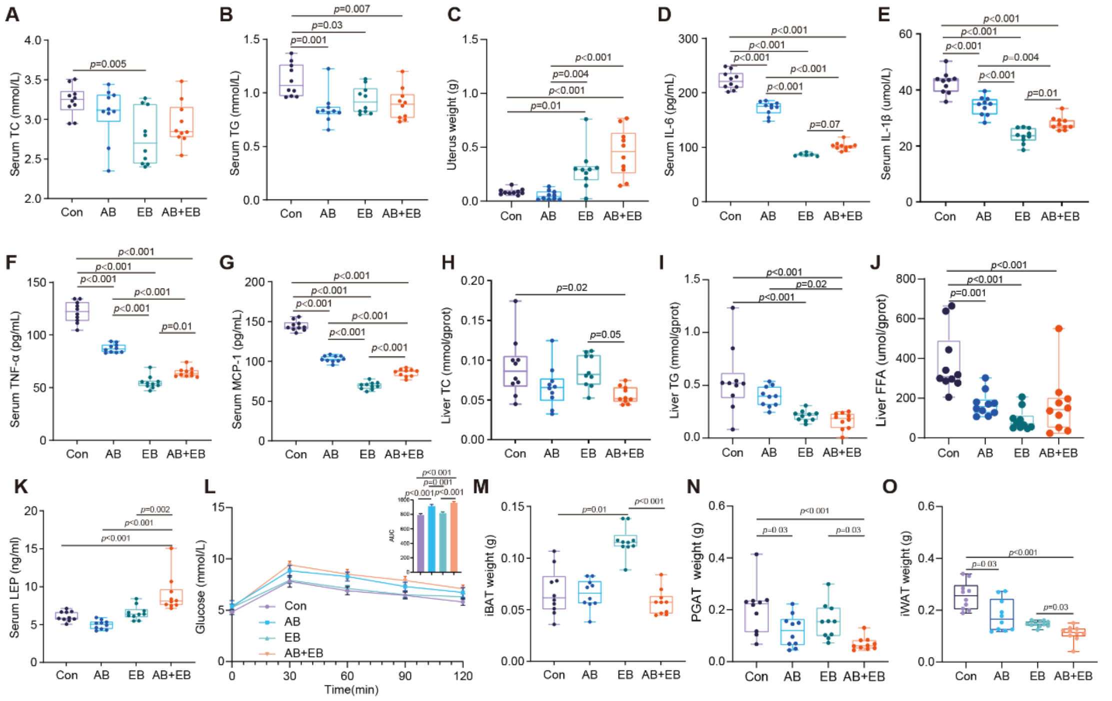

Figure S6. Gut microbiota alter the metabolic phenotype of EB supplemented OVX mice. (A-B) Serum cholesterol and triglyceride concentrations. (C) Weight of the uterus. (D-G) Serum levels of IL-6, IL-1β, TNF-α, MCP-1. (H-J) Concentrations of total cholesterol, triglyceride and free fatty acids in liver, respectively. (K) The concentration of serum leptin. (L) Blood glucose level curves during OGTT and AUC. (M-O) The weight of iBAT, PGAT, iWAT in four groups..
